# Editorial: Neuroprotection and Disease Modification in Parkinson’s Disease

**DOI:** 10.3389/fphar.2021.813471

**Published:** 2021-12-08

**Authors:** Matilde Otero-Losada, Paolo Gubellini, Francisco Capani, Santiago Perez-Lloret

**Affiliations:** ^1^ Centro de Altos Estudios en Ciencias Humanas y de la Salud, Universidad Abierta Interamericana, Consejo Nacional de Investigaciones Científicas y Técnicas, CAECIHS.UAI-CONICET, Buenos Aires, Argentina; ^2^ Aix-Marseille University, CNRS, IBDM UMR7288, Parc Scientifique de Luminy, Marseille, France; ^3^ Centro de Investigaciones en Psicología y Psicopedagogía (CIPP), Facultad de Psicología y Psicopedagogía, Pontificia Universidad Católica Argentina (UCA), Buenos Aires, Argentina

**Keywords:** Parkinson’s disease, neuroprotection, disease modification, experimental—animal models, disease progression

Parkinson’s disease (PD) is the second prevailing neurodegenerative disease in the world after Alzheimer’s. It affects about 1% of adults over 65 years old, with a growing incidence as the global population ages. Bradykinesia, tremor, and rigidity are the typical symptoms of PD. Yet, a myriad of non-motor symptoms like mood, sleep, and autonomic alterations impair patients’ life quality. Current treatments focus on PD symptoms but do not slow disease progression, and no preventing treatment is yet available against PD.

Identifying drugs and therapies that can change the course of PD is one of the most critical yet unmet needs in current pharmacological approaches to this disease. Past efforts have been ineffective in identifying these desired pharmaceuticals. Therefore, basic studies aiming at testing the potential neuroprotective properties of new molecules are needed.

This Research Topic focuses on addressing new pharmacological neuroprotective and disease-modifying strategies for PD, their target pathways, and their effects on humans. The goal is to contribute with updated information to developing new therapeutic strategies to prevent PD and halt its progression.

The topics covered are as follows:

*Acylated Ghrelin protects Against 6-OHDA-induced Neurotoxicity by Regulating Autophagic Flux* (He et al.). The authors show that ghrelin inhibits apoptosis and regulates autophagic flux thus protecting from 6-OHDA-induced neurotoxicity in rats and SH-SY5Y cells. Despite an action of autophagy activation, the neuroprotective effect of ghrelin is more reliant on restoration of TFEB level and relief of autophagic flux dysfunction. The importance of preserving functional autophagic flux against neurodegeneration is suggested, providing further basis for ghrelin as a potential drug for PD treatment (Jiang et al., 2008; Suda et al., 2018; He et al., 2018).

*A Ketone Ester Drink Enhances Endurance Exercise Performance in PD* (Norwitz et al.). Consumption of a ketone ester drink increased 24 ± 9% the time PD individuals sustained an 80-rpm cycling cadence compared with performance after drinking an isocaloric control beverage. Possible neuroprotective mechanisms are discussed. Ketone ester might synergize with exercise practice, holding potential as an indirect disease-modifying therapy in PD (Norwitz et al., 2019; Clarke et al., 2012).

*Contributive Role of TNF-α to* *l* *-DOPA-Induced Dyskinesia in a Unilateral 6-OHDA Lesion Model of Parkinson’s Disease* (Pereira et al.). Chronic l-dopa treatment induced a sustained glial inflammatory response, increasing the pro-inflammatory cytokines TNF-α and IL-1β in the striatum of 6-OHDA-lesioned dyskinetic mice. The antidyskinetic treatment combining capsazepine + cannabidiol prevented TNF-α production but not IL-1β in the dopamine-denervated striatum and glutamate-induced TNF-α release in astrocyte cultures. TNF-α release by glutamate-activated astrocytes may contribute to l-dopa-induced dyskinesia in a unilateral 6-OHDA PD lesion model, exacerbating corticostriatal glutamatergic input excitability and maintaining astrocytes activated via a self-reinforcing mechanism (Dos-Santos-Pereira et al., 2016; Del-Bel et al., 2016).

*Dl-3-n-Butylphthalide Alleviates Behavioral and Cognitive Symptoms Via Modulating Mitochondrial Dynamics in the A53T-α-Synuclein Mouse Model of Parkinson’s Disease* (Li et al.). The authors investigated whether Dl-3-n-butylphthalide (NBP), safe and effective in improving the non-tremor-dominant PD, could decrease dopaminergic neurons’ loss and α-synuclein deposition. NBP treatment partially preserved mitochondrial homeostasis by yet unknown mechanisms (Huang et al., 2018; Wang et al., 2016).

*Melatonin as a Chronobiotic and Cytoprotective Agent in Parkinson’s Disease* (Pérez-Lloret and Cardinali). The role of melatonin in PD prevention and treatment is discussed. Non-motor symptoms like hyposmia, rapid eye movement sleep behavior disorder (RBD), or depression may precede motor symptoms onset in PD for years and predict worse prognosis. Melatonin is cytoprotective and of mighty clinical usefulness in neurodegenerative disorders.

Daily bedtime administration of 3–12 mg of melatonin is effective in RDB treatment and might halt neurodegeneration to PD. Experimentally, melatonin curtailed PD symptomatology in doses, allometrically projected to humans, in the 40–100 mg/day range, rarely employed clinically. Double-blind, placebo-controlled clinical studies are needed to clarify and define melatonin neuroprotection (Cardinali 2019; Gilat et al., 2020).

*Endonasal CNS Delivery System for Blood-Brain Barrier Impermeant Therapeutic Oligonucleotides Using Heterotopic Mucosal Engrafting* (Pawar et al.). The blood-brain barrier (BBB) prevents 98% of all potential neuropharmaceuticals from reaching the brain. Brain derived neurotrophic factor (BDNF) has been reported to reverse PD progression. The authors investigated the distribution of BDNF AntagoNAT’s (BDNF AT’s), synthetic oligonucleotide-like compounds capable of upregulating endogenous BDNF expression, using an extra-cranial graft model in naïve rats using an innovative heterotopic mucosal engrafting technique. BDNF AT cationic liposomes (ideal size range 200–250 nm) were developed and characterized to enhance the delivery to rat brain. The delivered BDNF AT’s encapsulated in liposomes conferred neuroprotection in a rat 6-OHDA model of PD. (Pawar et al., 2018; Bleier et al., 2015).

*Edaravone Plays Protective Effects on LPS-Induced Microglia by Switching M1/M2 Phenotypes and Regulating NLRP3 Inflammasome Activation* (Li et al.). Inhibition of the NLRP3 inflammasome activation could protect dopaminergic neurons. The authors investigated the potential effects of edaravone on M1/M2 polarization of microglia in rats with dopaminergic damage induced by lipopolysaccharide (LPS). They found that edaravone improved neurobehavioral functions and played an anti-neuroinflammatory role in PD rats, possibly by inhibiting NLPR3 inflammasome activation and regulating microglia M1/M2 polarization (Gao et al., 2002; Liu et al., 2019).

## References

[B1] Bleier B. S. Kohman R. E. Guerra K. Nocera A. L. Ramanlal S. Kocharyan A. H. (2015). Heterotopic Mucosal Grafting Enables the Delivery of Therapeutic Neuropeptides across the Blood Brain Barrier. Neurosurgery 78, 448–457. 10.1227/NEU.0000000000001016 26352099

[B2] Cardinali D. P. (2019). Melatonin: Clinical Perspectives in Neurodegeneration. Front. Endocrinol. (Lausanne) 10, 480. 10.3389/fendo.2019.00480 31379746 PMC6646522

[B3] Clarke K. Tchabanenko K. Pawlosky R. Carter E. Todd King M. Musa-Veloso K. (2012). Kinetics, Safety and Tolerability of (R)-3-hydroxybutyl (R)-3-hydroxybutyrate in Healthy Adult Subjects. Regul. Toxicol. Pharmacol. 63, 401–408. 10.1016/j.yrtph.2012.04.008 22561291 PMC3810007

[B4] Del-Bel E. Bortolanza M. Dos-Santos-Pereira M. Bariotto K. Raisman-Vozari R. (2016). l-DOPA-induced Dyskinesia in Parkinson's Disease: Are Neuroinflammation and Astrocytes Key Elements? Synapse 70, 479–500. 10.1002/syn.21941 27618286

[B5] Dos-Santos-Pereira M. da-Silva C. A. Guimarães F. S. Del-Bel E. (2016). Co-administration of Cannabidiol and Capsazepine Reduces L-DOPA-Induced Dyskinesia in Mice: Possible Mechanism of Action. Neurobiol. Dis. 94, 179–195. 10.1016/j.nbd.2016.06.013 27373843

[B6] Gao H. M. Jiang J. Wilson B. Zhang W. Hong J. S. Liu B. (2002). Microglial Activation-Mediated Delayed and Progressive Degeneration of Rat Nigral Dopaminergic Neurons: Relevance to Parkinson's Disease. J. Neurochem. 81 (6), 1285–1297. 10.1046/j.1471-4159.2002.00928.x 12068076

[B7] Gilat M. Coeytaux Jackson A. Marshall N. S. Hammond D. Mullins A. E. Hall J. M. (2020). Melatonin for Rapid Eye Movement Sleep Behavior Disorder in Parkinson's Disease: A Randomised Controlled Trial. Mov. Disord. 35, 344–349. 10.1002/mds.27886 31674060 PMC7027846

[B8] He X. Yuan W. Li Z. Hou Y. Liu F. Feng J. (2018). 6-Hydroxydopamine Induces Autophagic Flux Dysfunction by Impairing Transcription Factor EB Activation and Lysosomal Function in Dopaminergic Neurons and SH-Sy5y Cells. Toxicol. Lett. 283, 58–68. 10.1016/j.toxlet.2017.11.017 29170033

[B9] Huang L. Wang S. Ma F. Zhang Y. Peng Y. Xing C. (2018). From Stroke to Neurodegenerative Diseases: the Multi-Target Neuroprotective Effects of 3-N-Butylphthalide and its Derivatives. Pharmacol. Res. 135, 201–211. 10.1016/j.phrs.2018.08.007 30103000

[B10] Jiang H. Li L. J. Wang J. Xie J. X. (2008). Ghrelin Antagonizes MPTP-Induced Neurotoxicity to the Dopaminergic Neurons in Mouse Substantia Nigra. Exp. Neurol. 212 (2), 532–537. 10.1016/j.expneurol.2008.05.006 18577498

[B11] Liu J. Jiang Y. Zhang G. Lin Z. Du S. (2019). Protective Effect of Edaravone on Blood-Brain Barrier by Affecting NRF-2/HO-1 Signaling Pathway. Exp. Ther. Med. 18 (4), 2437–2442. 10.3892/etm.2019.7859 31555355 PMC6755265

[B12] Norwitz N. G. Hu M. T. Clarke K. (2019). The Mechanisms by Which the Ketone Body D-β-Hydroxybutyrate May Improve the Multiple Cellular Pathologies of Parkinson's Disease. Front. Nutr. 6, 63. 10.3389/fnut.2019.00063 31139630 PMC6527784

[B13] Pawar G. N. Parayath N. N. Nocera A. L. Bleier B. S. Amiji M. M. (2018). Direct CNS Delivery of Proteins Using Thermosensitive Liposome-In-Gel Carrier by Heterotopic Mucosal Engrafting. PLoS One 13, e0208122–15. 10.1371/journal.pone.0208122 30517163 PMC6281301

[B14] Suda Y. Kuzumaki N. Sone T. Narita M. Tanaka K. Hamada Y. (2018). Down-regulation of Ghrelin Receptors on Dopaminergic Neurons in the Substantia Nigra Contributes to Parkinson's Disease-like Motor Dysfunction. Mol. Brain 11 (1), 6. 10.1186/s13041-018-0349-8 29458391 PMC5819262

[B15] Wang W. Karamanlidis G. Tian R. (2016). Novel Targets for Mitochondrial Medicine. Sci. Transl. Med. 8, 326rv3. 10.1126/scitranslmed.aac7410 PMC4819426 26888432

